# Optogenetic Activation of CA1 Pyramidal Neurons at the Dorsal and Ventral Hippocampus Evokes Distinct Brain-Wide Responses Revealed by Mouse fMRI

**DOI:** 10.1371/journal.pone.0121417

**Published:** 2015-03-20

**Authors:** Norio Takata, Keitaro Yoshida, Yuji Komaki, Ming Xu, Yuki Sakai, Keigo Hikishima, Masaru Mimura, Hideyuki Okano, Kenji F. Tanaka

**Affiliations:** 1 Department of Neuropsychiatry, School of Medicine, Keio University, Shinjuku, Tokyo, Japan; 2 Department of Physiology, School of Medicine, Keio University, Shinjuku, Tokyo, Japan; 3 Central Institute for Experimental Animals, Kawasaki, Kanagawa, Japan; 4 Department of Psychiatry, Graduate School of Medicine, Kyoto Prefectural University of Medicine, Kyoto, Japan; Indiana University, UNITED STATES

## Abstract

The dorsal and ventral hippocampal regions (dHP and vHP) are proposed to have distinct functions. Electrophysiological studies have revealed intra-hippocampal variances along the dorsoventral axis. Nevertheless, the extra-hippocampal influences of dHP and vHP activities remain unclear. In this study, we compared the spatial distribution of brain-wide responses upon dHP or vHP activation and further estimate connection strengths between the dHP and the vHP with corresponding extra-hippocampal areas. To achieve this, we first investigated responses of local field potential (LFP) and multi unit activities (MUA) upon light stimulation in the hippocampus of an anesthetized transgenic mouse, whose CA1 pyramidal neurons expressed a step-function opsin variant of channelrhodopsin-2 (ChR2). Optogenetic stimulation increased hippocampal LFP power at theta, gamma, and ultra-fast frequency bands, and augmented MUA, indicating light-induced activation of CA1 pyramidal neurons. Brain-wide responses examined using fMRI revealed that optogenetic activation at the dHP or vHP caused blood oxygenation level-dependent (BOLD) fMRI signals *in situ*. Although activation at the dHP induced BOLD responses at the vHP, the opposite was not observed. Outside the hippocampal formation, activation at the dHP, but not the vHP, evoked BOLD responses at the retrosplenial cortex (RSP), which is in line with anatomical evidence. In contrast, BOLD responses at the lateral septum (LS) were induced only upon vHP activation, even though both dHP and vHP send axonal fibers to the LS. Our findings suggest that the primary targets of dHP and vHP activation are distinct, which concurs with attributed functions of the dHP and RSP in spatial memory, as well as of the vHP and LS in emotional responses.

## Introduction

The hippocampus has been extensively studied, and implicated in memory, cognition, and emotion [1]. Lesion studies have proposed a functional dissociation along the dorsoventral axis of the hippocampus [2] by showing that the dorsal hippocampus (dHP) plays a crucial role in spatial learning [3], whereas the ventral hippocampus (vHP) is involved in emotional responses [4] (but see [5,6] for other possibility). Electrophysiological activity is also distinct within the hippocampus along the longitudinal axis [7]. However, to our knowledge, comparison of extra-hippocampal influences stemming from dHP and vHP activity has not yet been performed. Although anatomical studies have revealed overlapping but different patterns of axonal outputs from the dHP and vHP [8,9], it is unclear whether the extra-hippocampal influence of dHP and vHP activity faithfully reflects the density of these anatomical connections. Additionally, comprehensive electrical recording from downstream targets of the hippocampus is impractical using standard electrophysiological methods because the CA1 region, being the main output area of the hippocampus, sends axonal outputs to over 50 areas in the brain [9]. Further, selective hippocampal activation has been difficult until recently using conventional electrical stimulation, since this might stimulate non-hippocampal cells that send their axons within the hippocampus.

Recent pioneering work by Lee et al [10] has combined optogenetics with functional magnetic resonance imaging (ofMRI), thereby allowing measurements of brain-wide responses after optogenetic activation of selective cortical and thalamic neuronal populations expressing the light-gated cation selective membrane channel channelrhodopsin-2 (ChR2). Recently, we developed a bigenic mouse, whose CA1 pyramidal neurons express a step-function opsin variant, ChR2(C128S), channels of which could be opened or closed with blue and yellow light illumination, respectively [11]. Here, we used the transgenic mouse model and ofMRI measurements to compare the spatial distribution of brain-wide responses of blood oxygenation level dependent (BOLD) fMRI signals upon optogenetic stimulation of CA1 pyramidal neurons at the dHP or vHP. Individual connection strength from the dHP and vHP to corresponding downstream targets was also estimated using the amplitude of BOLD signal responses.

## Materials and Methods

### Ethics Statement

All animal experiment procedures were carried out in accordance with the guidelines of the Japanese Neuroscience Society. The protocol was approved by the Keio University and CIEA Animal Care and Use Committee (#12034).

### Surgery for optical fiber implantation

**Fig 1:**
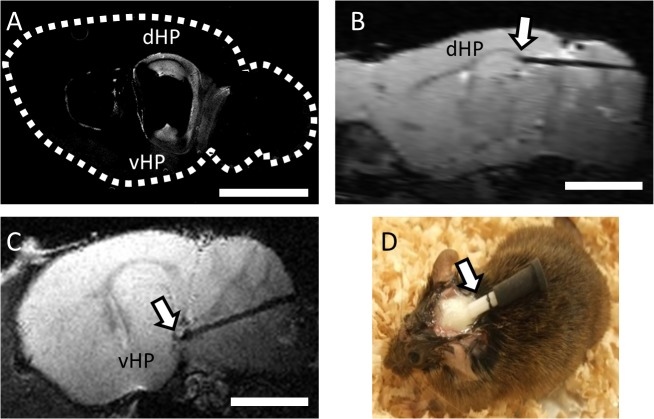
An implanted optical fiber targeted at the dorsal or ventral hippocampus in a transgenic mouse. **A,** Expression of ChR2(C128S) at CA1 pyramidal neurons at the dHP and vHP of a transgenic mouse is demonstrated by immunostaining against EYFP on a sagittal section at lateral −2.8 mm. A dotted white line depicts the contour of the brain with the rostral side on the left. **B, C,** Representative anatomical T2-weighted images showing an implanted optical fiber targeting the dHP (**B**) or vHP (**C**) seen as a dark shadow track. The arrow indicates the tip of the optical fiber. In **C**, the brain section is rotated 12.0 degrees laterally from the sagittal plane to depict the full length of the fiber. **D,** A photograph of a transgenic mouse implanted with a fiber optic cannula (shown with an arrow). The cannula is covered with a rubber cap. Scale bars in A–C: 3 mm.

Double transgenic mice (Htr5B-tTA::tetO-ChR2(C128S)-EYFP; 16 males and 13 females from 8–12 weeks old) were used [11]. Animals were anesthetized with sevoflurane (1.5∼3%, 0.25 mL/min). An optical fiber was horizontally inserted to penetrate through the cerebellum and to target the left dHP or vHP (Fig. 1B,C; S1 Fig.). Specifically, to illuminate CA1 pyramidal neurons at the dHP, a craniotomy (Ø 1 mm), centered at 7.9 mm posterior to the Bregma (AP −7.9 mm), 1.8 mm lateral from the midline to the left (ML −1.8 mm), and 2.1 mm ventrally from Bregma-lambda line (DV +2.1 mm) was performed at the occipital skull for the insertion of an optical fiber (Ø 200 or 300 μm; CFML12L05 or CFMC13L05, Thorlabs), which was inserted at a depth of 5 mm with an 11.5-degree elevation angle through the craniotomy opening. For illumination at the vHP, through a craniotomy (AP −8.7, ML −2.0, DV +3.5 in mm), an optical fiber was inserted at a 4.77 mm depth with a 7.64-degree depression angle and with an azimuth of 16.7 degrees laterally. The side of an optical fiber was painted in black to avoid stray light. After covering the exposed skull with dental acrylic (Super-Bond C&B, Sun Medical), the animal was returned to its homecage for recovery for more than 1 week (Fig. 1D). The optical fiber insertion through the cerebellum was necessary because a CryoProbe, a mouse specific MRI detector [12], occupies the dorsal space above the skull. For acute electrophysiological experiments, additional craniotomies (Ø 1.5 mm) for electrode insertion into the dHP or vHP were prepared at AP −2.0 and ML −1.4, or AP −3.3 and ML −3.0 in mm, respectively.

### ofMRI in anesthetized mice

Animals with an optical fiber implantation were anesthetized using medetomidine (0.3 mg/kg s.c. bolus injection followed by infusion at 0.6 mg/kg/h) according to Adamczak et al [13]. Body temperature (37.0 ± 0.5°C) and the respiration rate (110–130 breaths/min) were continuously monitored (SAM PC monitor v6.17, SAII).

Structural and functional MRI was performed using a 7.0-Tesla MRI apparatus equipped with actively shielded gradients at a maximum strength of 700 mT/m (Biospec 70/16, Bruker BioSpin) with CryoProbe (Bruker BioSpin AG) [14]. Structural T2-weighted images were acquired using a rapid acquisition process with a relaxation enhancement (RARE) sequence in coronal orientations (repetition time [TR], 6100 ms; echo time [TE], 48 ms; spectral bandwidth [BW], 5 kHz; RARE factor, 8; number of averages, 4; number of slices 52; spatial resolution, 75 × 75 × 300 μm). Before fMRI measurement, a field map was acquired to reduce signal loss artifacts (TE, 1.520 ms; 5.325 ms; TR, 20 ms; spatial resolution, 300 × 300 × 300 μm; matrix, 64 × 64 × 64 voxels). fMRI was performed using a gradient-echo echo-planar sequence (TR, 1500 ms; TE, 20 ms; BW, 250 kHz; flip angle, 50°; number of segments, 2; number of averages 1; number of slices 18; spatial resolution, 200 × 200 × 500 μm; FOV, 19.2 × 19.2 mm; matrix, 96 × 96 × 18 voxels). This functional image covered the whole brain except the olfactory bulb and the cerebellum. Total scanning time was 5 min (200 volumes with a 1.5-s interval).

Blue and yellow light pulses (0.5-s duration, 1.1∼2.5 mW at the tip of an optical fiber; LEDC2-B/A, Doric) were delivered with 30-s intervals in order to open and close cation selective membrane channels of ChR2(C128S), respectively.

### Multichannel extracellular recordings

**Fig 2:**
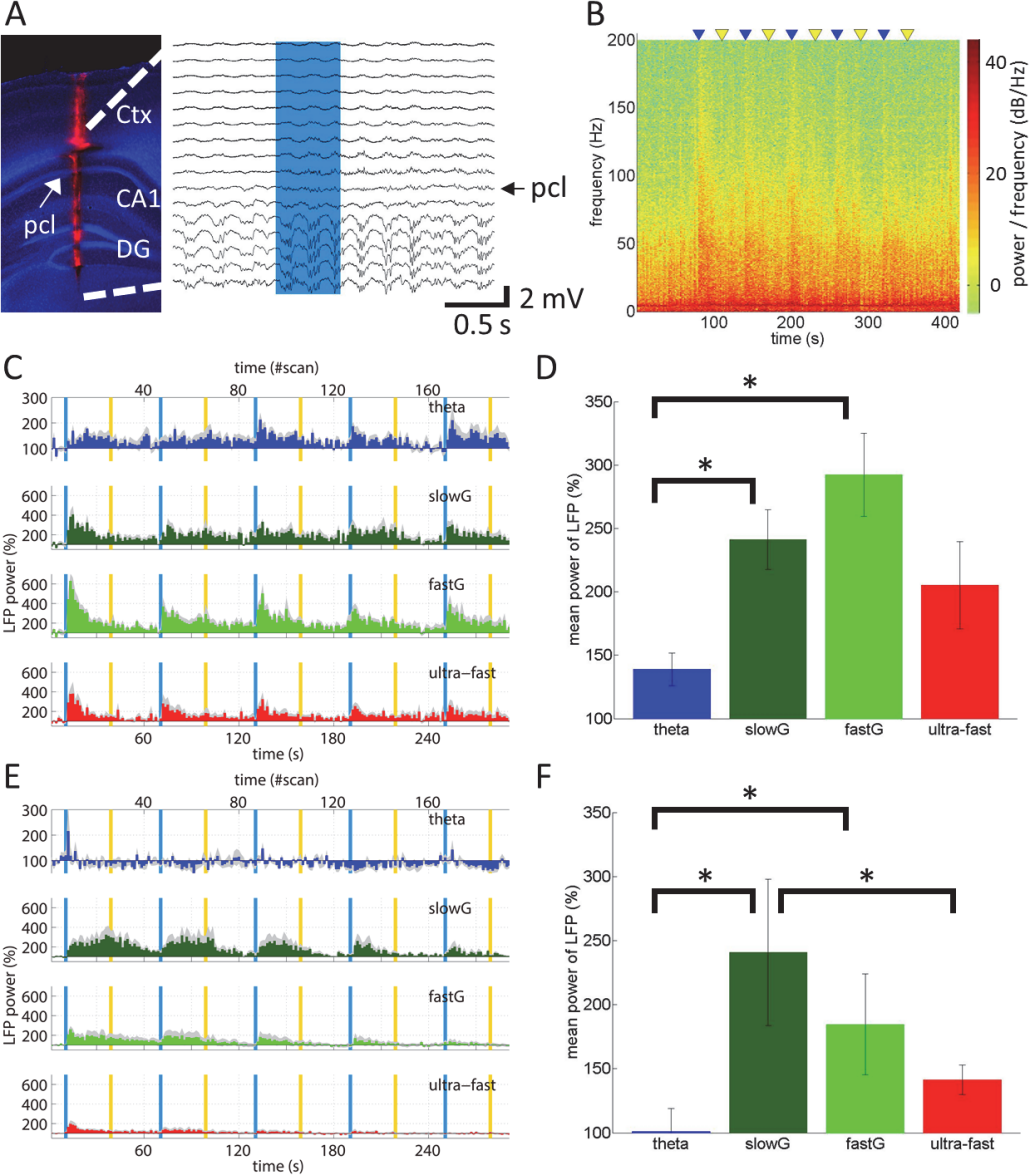
Local field potential responses upon optogenetic stimulation of CA1 pyramidal neurons. **A,** (left panel) A 16-channel silicon probe electrode is inserted to measure LFPs in an anesthetized transgenic mouse. After tissue fixation, brain sections are counterstained with DAPI (blue). The electrode track is identified with DiI fluorescence (red). The arrow indicates the pyramidal cell layer (*pcl*) of the CA1 region. Ctx, cortex; DG, dentate gyrus. (right panel) Representative LFP traces measured simultaneously at different depths of the cortex and the hippocampus. The blue region indicates the period of blue-light illumination for 0.5 s used to stimulate ChR2(C128S). Note that the light illumination affected LFP not only at the ChR2(C128S)-expressing CA1 *pcl*, but also at the DG where ChR2(C128S) is not expressed. Also note that fluctuations of several LFP traces at DG before blue-light illumination are spontaneous theta waves. **B,** A representative LFP spectrogram at the *pcl* of the CA1 region is shown. Blue and yellow triangles indicate the delivery of blue and yellow light pulses with 0.5-s durations used to activate and deactivate ChR2(C128S), respectively. Light pulses were separated by 30 s and repeated 5 times every 1 min. The color bar indicates power spectral densities of LFP. **C, E,** The time course of LFP-power at each frequency band, i.e., theta (blue), slow gamma (slowG; green), fast gamma (fastG; light green), and ultra-fast (red) bands, are normalized to the pre-stimulus period. The x-axis at the top corresponds to fMRI scan counts. Note that upper limit of the y-axis for theta is smaller to improve visibility. Gray shading in each time course indicates the SEM. Optical stimulation and electrophysiological recording was performed at dorsal (C) and ventral (E) hippocampus. **D, F,** Comparison of the LFP mean power at each frequency band during the first activation period (9∼39 s), which was normalized to the pre-stimulus period. The LFP at the gamma band is augmented as compared to the other frequency bands upon optogenetic activation of CA1 pyramidal neurons. Results are obtained at the dorsal (D) and ventral (F) hippocampus.

In a separate group of experiments, 16-channel extracellular recordings were made from anesthetized animals (urethane 1.6 g/kg, i.p.) using a linear silicon probe (100-μm spacing, 177-μm^2^ recording site area; NeuroNexus Technologies), which was inserted at a depth of 1.85 or 5.0 mm ventrally from the pia, for recording from the dHP or vHP, respectively. Proximity to the hippocampal pyramidal cell layer was judged by (1) the depth of the probe, (2) the presence of action potential discharges, and (3) the phase reversal of the local field potential (LFP) at theta frequencies above and below the recording site. A silicon probe was coated with a lipophilic fluorescent dye DiI (D-282, Invitrogen) (Fig. 2A). Electrophysiological signals were recorded with a RZ2 neurophysiology workstation (Tucker-Davis Technologies).

### Multi unit activity (MUA) recording using an optrode

**Fig 3:**
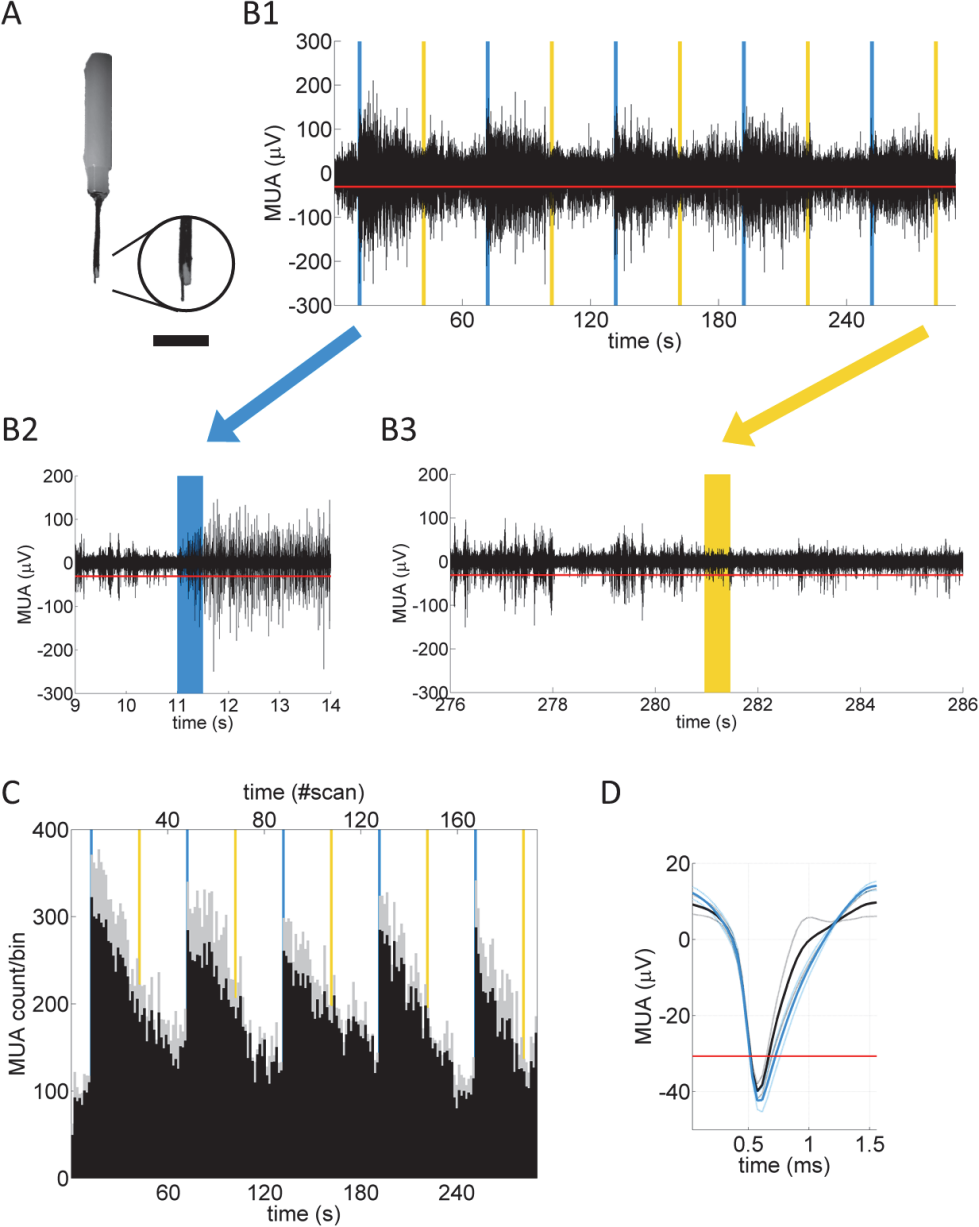
Multi unit activities (MUA) at CA1 pyramidal cell layer upon optogenetic activation. **A,** A photograph of a custom-made optrode, which was inserted vertically to record MUA at CA1 pyramidal cell layer of dHP of an anesthetized transgenic mouse. An expanded view of a tip of an optrode is shown in a circle at the right. A tungsten-wire was protruded ∼0.5 mm from a tip of an optical fiber, which corresponds to the distance between a tip of an optical fiber and illumination target in the hippocampus in fMRI experiments (see Fig. 1 B,C; S1 Fig.). Also note that a side of an optical fiber was painted in black to reduce stray light. Scale bar: 3.5 mm for a whole view, 1 mm for an expanded view. **B,** A representative time course of MUA activities. Vertical lines of blue and yellow indicate 0.5 s-illumination of light of each color, respectively. **B2** and **B3** show an expanded view of MUA upon blue and yellow light illumination, respectively. Note that in **B1**, vertical lines in blue and yellow are drawn thicker than 0.5 s for visibility purpose. **C,** MUA count per 1.5 s bin was obtained from 6 recording sessions from 3 transgenic animals. The x-axis at the top corresponds to fMRI scan counts. Gray shading indicates the SEM. Vertical lines in blue and yellow indicate bins when light in each color was illuminated at dHP. **D,** Mean traces of MUA during pre-stimulus period (0∼11 s) and the first activated period (11∼41 s) are shown in black and blue, respectively, obtained from 6 recording sessions from 3 transgenic animals.

MUA at CA1 pyramidal cell layer of the dorsal hippocampus (AP −2.0 and ML −1.4 in mm) of an urethane anesthetized transgenic mouse was measured using a custom made optrode, which is consisted of a tungsten-wire (Ø 50 μm, #2016971, California Fine Wire) glued to an optical fiber (Ø 200 μm, CFML12L05, Thorlabs) (Fig. 3A).

### Data processing and analysis

ofMRI data analysis was performed using SPM8 software (http://www.fil.ion.ucl.ac.uk/spm). This consisted of head movement correction, adjustments of acquisition timing across slices, and smoothing using a Gaussian kernel of 0.4-mm full width at half maximum. Structural and functional images were spatially normalized to a standard structural brain averaged from 20 C57BL/6 mice. SPM *t*-contrast map was obtained with a threshold value (uncorrected p < 0.005 with a minimum cluster size > 10 voxels) using a design matrix consisted of pre-activated and optogenetically activated periods. Specifically, pre-activation corresponds to 9 s periods just before blue-light illumination (e.g. blue vertical lines in Fig. 2C), and optogenetically activated periods are 30 s periods between blue and yellow light illumination (e.g. pairs of blue and yellow vertical lines in Fig. 2C). Thus, among 200 volumes in a single fMRI measurement, pre-activation and optogenetically activated periods correspond to fMRI-scan numbers of #1∼6, 40∼45, 80∼85, 120∼125, 160∼165 and #7∼26, 47∼66, 87∼106, 127∼146, 167∼186, respectively. We performed fixed effect analysis on 5 (dHP) or 7 (vHP) animals using a design matrix contrasting BOLD signal amplitudes at each voxel during the pre-activation vs. optogenetically activated periods. The uncorrected p-value threshold of 0.005 and a cluster size of 10 were objectively chosen via Monte Carlo simulations using the “AlphaSim” implementation in a toolbox REST [15], resulting in a multiple comparisons corrected type I error of 5%. The parameters used for the simulations were as follows: full width at half maximum, 0.4 mm; cluster connection radius, 0.2 mm; individual voxel threshold probability, 0.005; number of Monte Carlo iterations, 100,000; voxels in mask, 8212.

**Fig 4:**
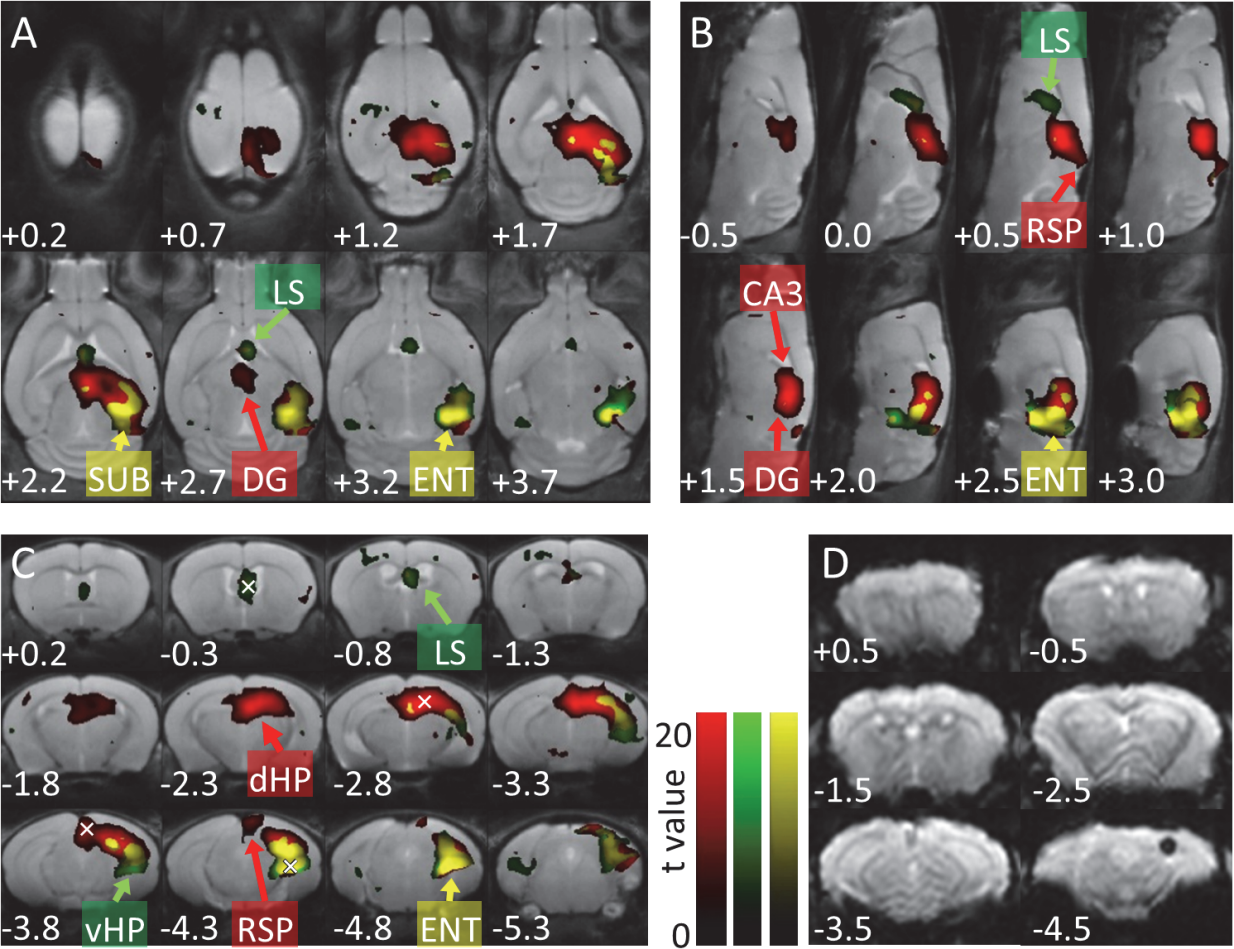
Distinct brain-wide BOLD responses upon optogenetic activation of dorsal or ventral CA1 pyramidal neurons. Activation *t*-maps overlaid on T2-weighted standard structure images, showing spatial distributions of BOLD signals in response to optogenetic activation of CA1 pyramidal neurons at the dHP (red) or vHP (green). Yellow indicates regions that responded to both dHP and vHP activation. Values at the lower left indicate dorso-ventral depth (**A**), medio-lateral distance (**B**), or anterior-posterior (AP) distance (**C, D**) in mm. Within the hippocampal formation, optogenetic activation at both the dHP and vHP results in BOLD responses at the CA1, CA3, dentate gyrus (DG), subicular region (SUB), and entorhinal cortex (ENT). Outside the hippocampal formation, BOLD responses in the retrosplenial cortex (RSP) are evoked only by dHP activation. In contrast, fMRI signals at the lateral septum (LS) are induced only by vHP activation. Color bars indicate *t*-values ranging from 0 (dark) to 20 (bright). White x-marks at AP −0.3, −2.8, −3.8, and −4.3 in (**C**) indicate approximate location of ROIs for BOLD-timecourses of LS, dHP, RSP, and vHP, respectively. (**D**) Example of raw functional images before normalization from a transgenic mouse (animal ID: SNT025). The black hole at AP −4.5 mm indicates an optical fiber.

**Fig 5:**
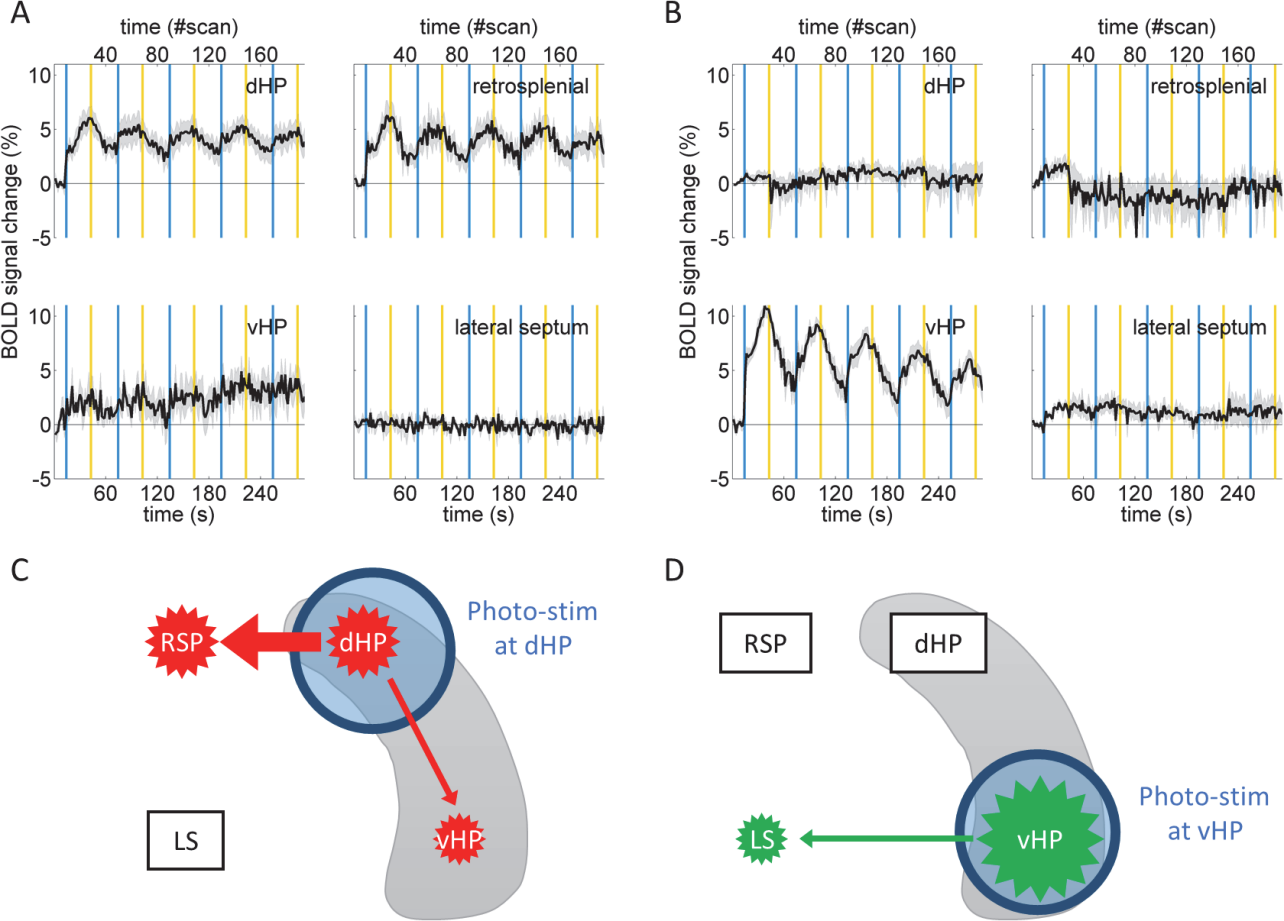
BOLD signal amplitudes upon optogenetic activation at the dorsal or ventral hippocampus. Repeated fMRI signal responses are observed upon optogenetic activation of CA1 pyramidal neurons at the dHP (**A**) or vHP (**B**). dHP activation evokes BOLD responses at the dHP, retrosplenial cortex (RSP), and vHP (**A**), whereas vHP activation evokes responses at the vHP and lateral septum (LS) (**B**). Pairs of blue and yellow vertical lines indicate periods of optogenetic activation. The x-axis at the top shows to the scan number of fMRI measurements. Grey shading indicates the SEM. **C, D,** Summary of BOLD responses upon optogenetic activation at dHP and vHP, respectively. The size of star polygons corresponds to magnitude of BOLD responses upon optogenetic stimulation at dHP (**C, red**) or vHP (**D, green**). Thickness of arrows suggests influence of dHP or vHP activation. Note that BOLD response at subiculum and entorhinal cortex, which are part of the hippocampal formation, is not included in this schematic picture.

Nomenclature of brain regions was assigned by comparing a standard structure image, the Allen Mouse Brain Atlas (Brain Explorer 2, v2.3.3), and the stereotaxic brain map [16]. Timecourses of BOLD signals at each brain region (dHP, vHP, RSP, and LS) were obtained with a spherical ROI of 0.5-mm diameter (10 voxels), using the SPM toolbox MarsBar (http://marsbar.sourceforge.net). Spatial coordinates of these ROIs in a standard structure image were (AP, ML, DV in mm): −3.1, −1.9, 1.8 for dHP; −4.4, −3.1, 3.6 for vHP; −3.5, −0.2, 1.2 for RSP; −0.4, −0.3, 2.8 for LS. The same ROIs were applied to spatially normalized functional images of all animals. Approximate location of these ROIs are indicated as white x-marks in Fig. 4C. ROIs for dHP and vHP were placed near dorsal or ventral poles of the hippocampus, respectively, to avoid ambiguity of the boundary between dHP and vHP. Comparison of BOLD response amplitudes between brain regions was performed using the peak amplitude of BOLD signal changes upon the first optogenetic stimulation, i.e. periods between the first blue and yellow light illumination (see Fig. 5A,B).

Electrophysiological data analysis was performed using MATLAB (R2013a, MathWorks). LFPs were decomposed to frequency bands of theta (4–8 Hz), slow gamma (30–45 Hz), fast gamma (55–90 Hz), and ultra-fast oscillations (140–200 Hz) [17]. LFP power at each band was binned to 1.5 s, which corresponded to fMRI imaging intervals. In a measurement using an optrode, MUA was extracted by high-pass finite impulse response (FIR) filtering (> 300 Hz) LFP-signals with a threshold set at −30.7 μV (shown in red line in Fig. 3B,D), which was obtained from 4σ where σ = median{absolute value of the filtered LFP / 0.6745} [18]. Count of MUA was binned to 1.5 s, which corresponds to fMRI imaging intervals.

Light power at lateral septum (LS) upon illumination at the vHP was estimated using a model based on direct measurements in mammalian brain (http://web.stanford.edu/group/dlab/cgi-bin/graph/chart.php) using parameters: light wavelength 473 nm; numerical aperture of a fiber 0.39; light power from a fiber tip 2.5 mW; fiber core radius 0.1 mm, distance from a tip of an optical fiber to LS ∼2 mm.

Data are expressed as mean ± SEM across animals. Two-tailed *t*-tests were used for comparisons of two population means, unless otherwise noted.

### Histology

After measurements, animals were deeply anesthetized and transcardially perfused with PB containing 4% PFA. To verify electrode tracks, coronal sections were cut at 200-μm thickness using a vibratome (Pro-7 Linear Slicer, DSK) and DiI fluorescence was examined (Fig. 2A). Immunohistochemistry of EYFP to confirm hippocampal expression of ChR2(C128S)-EYFP and *in situ* hybridization to examine *c-fos* mRNA expression were performed as previously [11].

## Results

Twenty-nine transgenic mice expressing ChR2(C128S) at the CA1 pyramidal neurons (Fig. 1A) were used (14 and 15 animals for electrophysiology and ofMRI, respectively). Light illumination at the dHP or vHP was achieved by inserting an optical fiber (Fig. 1B,C; S1 Fig.). Blue- and yellow-light illumination was used to open and close a non-selective cation channel of a step-function opsin ChR2(C128S), respectively (e.g. triangles in Fig. 2B top, vertical lines in Fig. 2C,E).

### Optogenetic stimulation of ChR2(C128S)-expressing CA1 pyramidal neurons increases the local field potential power and multi unit activities *in vivo*

A linear silicon probe was inserted into the dHP of a transgenic mouse to measure LFP responses upon optogenetic stimulation of the ChR2(C128S)-expressing CA1 pyramidal neurons at the dHP (Fig. 1B and Fig. 2A left panel, n = 5 mice). Representative LFP traces (Fig. 2A right, expanded view at S2A Fig.) show the immediate responses upon stimulation at the hippocampal CA1, DG, and cortex. Note that activation of ChR2(C128S) persists after cessation of blue-light illumination, until yellow-light illumination. Spectrum analysis of LFP responses at the CA1 pyramidal cell layer (*pcl*) indicates wide-band frequency responses upon the optogenetic stimulation (Fig. 2B, expanded view at S2B Fig.).

Time courses of LFP power at the *pcl* of the CA1 region demonstrate that the optogenetic stimulation significantly augments LFP power at representative frequency bands in the hippocampus, i.e., theta, gamma, and ultra-fast (Fig. 2C) [19]. LFP power at the gamma and ultra-fast frequency bands was strongly increased at the beginning of the stimulation, followed by a gradual decrease even within the 30-s period of channel opening of ChR2(C128S) (periods between a pair of blue and yellow vertical lines in Fig. 2C). Among these frequency bands, the gamma frequency was most increased upon optogenetic stimulation. Indeed, average power of the LFP at the slow and fast gamma frequencies during the first light activation period (10∼40 s in Fig. 2C), which was normalized to the pre-stimulus period, was significantly higher than that at the theta oscillations (F(3, 12) = 4.7, p < 0.05, two-way ANOVA followed by Tukey’s LSD test; theta 139 ± 14%, slow gamma 241 ± 26%, fast gamma 292 ± 37%, ultra-fast 205 ± 38%; Fig. 2D). LFP recordings from the vHP with optogenetic stimulation at the vHP (Fig. 1C) also resulted in augmentation of LFP power at the gamma and ultra-fast frequency bands, although its time course was different from that of dHP (F(3, 15) = 4.5, p < 0.05, two-way ANOVA followed by Tukey’s LSD test; theta 101 ± 20%, slow gamma 241 ± 63%, fast gamma 184 ± 43%, ultra-fast 141 ± 13%, n = 6 mice, Fig. 2E, F). Moreover, enhancement of LFP power at theta frequency band was not observed in the case of vHP.

To further analyze responses of ChR2(C128S)-expressing CA1 pyramidal neurons, MUA at dHP upon light illumination at dHP was measured using an optrode (Fig. 3A, n = 3 mice). Mean MUA counts per 1.5 s bin during an optogenetically activated period was significantly augmented than that during pre-activation period (F(5,10) = 9.8, p < 0.05, two-way ANOVA followed by Tukey’s HSD test; MUA count/bin: pre-activation period 89 ± 8 vs. 1^st^ ∼ 5^th^ activation period 256 ± 49, 237 ± 53, 217 ± 34, 230 ± 26, 185 ± 42, respectively, Fig. 3C). The shape of mean MUA traces during pre-activation (black trace in Fig. 3D) and the first activation period (blue trace in Fig. 3D) seemed similar (Fig. 3D). Indeed, the negative peak amplitude of these traces was not different significantly (-39.9 ± 1.9 μV vs. −42.8 ± 2.8 μV during pre-activation (black trace) and the first activation (blue trace) period, respectively. p = 0.44, n = 3 mice). Note that the above electrophysiological data suggest the absence of seizure activities in the hippocampus upon the optogenetic stimulation, which is further supported by lack of *c-fos* mRNA expression after fMRI measurements (S6 Fig.) [20].

### Optogenetic activation at the dHP or vHP evokes distinct spatial distribution of brain-wide fMRI responses

ofMRI was performed to compare the spatial distributions of brain-wide BOLD responses upon optogenetic activation of CA1 pyramidal neurons at the dHP or vHP of an anesthetized transgenic mouse (5 and 7 mice for dHP and vHP, respectively). The procedure for the blue- and yellow-light illumination was the same as in electrophysiological experiments (Fig. 2).

Fig. 4 summarizes the spatial distribution of BOLD responses upon optogenetic activation at the dHP (red) or vHP (green), respectively. Brain regions that responded to both dHP and vHP activation are shown in yellow. Optogenetic activation of CA1 pyramidal neurons at the dHP or vHP induced BOLD responses *in situ* (dHP and vHP in Fig. 4C, respectively).

Within the hippocampal formation, BOLD responses were also observed at the subicular complex (SUB in Fig. 4A) and entorhinal cortex (ENT in Fig. 4A∼C), which receive dense axonal fibers from the CA1. In addition, the CA3 and DG, both of which have no direct axonal connection from the CA1 pyramidal neurons, showed BOLD responses (CA3 and DG in Fig. 4B). BOLD signals at the DG coincided with LFP responses in the DG upon optogenetic activation of CA1 pyramidal neurons (Fig. 2A representative traces). Optogenetic activation at the dHP resulted in BOLD responses at the vHP (hereafter written as B(vHP; dHP) to indicate BOLD response(s) at the vHP induced upon optogenetic activation of the dHP). In fact, vHP is mostly yellow in Fig. 4C). BOLD responses at the dHP upon optogenetic stimulation of the vHP, B(dHP; vHP), were not observed (dHP is red in Fig. 4C). These observations were supported by a calculation of the spatial volume of BOLD responses in the hippocampus (HP) upon dHP or vHP activation. Voxel counts of the B(HP; dHP) were significantly higher than those of the B(HP; vHP) (113 ± 15 vs 56 ± 12 voxels, p = 0.014, n = 5 and 7 mice, respectively). Note that the amplitude of the B(dHP; dHP) was lower than that of the B(vHP; vHP) (see next section; Fig. 5A upper left vs 5B lower left). Also note that more ventral portion of the hippocampus was activated only upon vHP activation (green area in Fig. 4C, AP −3.8 mm).

Outside the hippocampal formation, prominent BOLD responses were observed at the retrosplenial cortex (RSP) only upon dHP activation (RSP in Fig. 4C). The lateral septum (LS) responded only to vHP activation (LS in Fig. 4A,B). Representative images of raw BOLD signals are shown at Fig. 4D. Three-dimensional statistical map contrasting dHP- vs. vHP-responses are shown at S3 Fig. Note that in Fig. 4, statistical maps contrast pre-activation vs. activated periods of dHP and vHP, respectively.

### BOLD signals upon dHP or vHP activation show different response amplitudes

BOLD signal response amplitudes upon optogenetic stimulation at the dHP or vHP were compared in different brain regions using peak values of BOLD signals during the first stimulation period, i.e., a period between the first pair of blue and yellow vertical lines in each time course (Fig. 5A,B, expanded view at S4 Fig.). For multiple comparisons of BOLD signal amplitude, one-way ANOVA (F(8, 46) = 14.0, p < 0.001) with nine levels followed by Tukey’s LSD test was employed. The nine levels include signal amplitudes of B(dHP; dHP), B(RSP; dHP), B(LS; vHP), and so forth.

Within the hippocampus, peak amplitude of B(vHP; vHP) was greater than that of the B(dHP; dHP) (11.6 ± 0.9% vs 6.9 ± 1.2%, p < 0.05, n = 7 and 5 mice, respectively; Fig. 5B lower left vs 5A upper left). The presence of the B(vHP; dHP) signal and absence of the B(dHP; vHP) signal are denoted in the time courses of BOLD signals (Fig. 5A lower left and 5B upper left) in accordance with spatial distribution of BOLD responses (Fig. 4C vHP and dHP). Indeed, the peak amplitude of the B(vHP; dHP) was significantly larger than that of the B(dHP; vHP) (5.5 ± 1.9% vs 2.0 ± 0.4%, p < 0.05, n = 5 and 7 mice, respectively. Note these values are mean of peak values of each animal, while traces in Fig. 5A,B show average traces regardless of their peak timing).

Outside the hippocampal formation, the peak amplitude of the B(RSP; dHP) was comparable to that of the B(dHP; dHP) (6.9 ± 1.5% vs 6.9 ± 1.2%, n = 5 mice. Fig. 5A upper right vs left). In contrast, the peak amplitude of the B(LS; vHP) was significantly lower than that of the B(vHP; vHP) (3.7 ± 1.0% vs 11.6 ± 0.9%, p < 0.05, n = 7 mice, Fig. 5B lower left vs right). Nevertheless, the peak amplitude of the B(LS; vHP) was significantly higher than the peak amplitude of BOLD signal during the pre-stimulus period (3.7 ± 1.0% vs 0.9 ± 0.2%, p < 0.05, n = 7 mice, Fig. 5B lower right) in accordance with spatial distribution of B(LS; vHP) (Fig. 4A∼C).

We also compared peak values of B(dHP; dHP) induced upon reduced light power (S5A,B Fig.). Only 100% light power illumination, which was used in Figs. 2∼5, induced significant increase of mean BOLD signals during an activation period (F(3,8) = 8.6, p < 0.01, one-way ANOVA followed by Tukey’s HSD test, peak amplitude of BOLD signal during pre-activation, 1.6 ± 0.8%; activation with 10% light power, 2.3 ± 0.8%; 20% light power, 2.8 ± 0.5%; 100% light power, 8.6 ± 1.8%; n = 3 animals, S5B Fig.). In addition, illumination of only yellow light did not modulated BOLD signal fluctuations (n = 2 animals, S5C Fig.). The blue- and yellow-light illumination did not induce BOLD response in WT mice (n = 3 animals, S5D Fig.).

## Discussion

By performing ofMRI of the transgenic mice, we compared brain-wide responses upon optogenetic activation of CA1 pyramidal neurons at the dHP or vHP. Distinct spatial patterns of BOLD responses were observed upon stimulation of the dHP or vHP. Additionally, connection strength from the dHP or vHP to other brain areas was estimated based on the magnitude of BOLD signal change. A strong influence of dHP activation on the RSP was found in terms of BOLD responses [21]. These observations are summarized and schematized in Table 1 and Fig. 5C,D.

**Table 1:** Summary of anatomical distribution of BOLD responses.

Target of optogenetic stimulation	BOLD signal response			
	dHP	vHP	RSP	LS
dHP	++ (6.9 ± 1.2)	+ (5.5 ± 1.9)	++ (6.9 ± 1.5)	− (2.3 ± 0.3)
vHP	− (2.0 ± 0.4)	+++ (11.6 ± 0.9)	− (3.6 ± 0.8)	+ (3.7 ± 1.0)

Amplitude of BOLD responses at each brain area upon optogenetic stimulation at dorsal or ventral hippocampus (dHP or vHP) was compared with + (smallest) ∼ +++ (largest). ‘–‘ indicates no response confirmed by spatial distribution of BOLD responses (Fig. 4). Values in parenthesis show peak amplitude ± SEM (%) of BOLD signals during the first stimulation period (n = 5 and 7 animals for stimulation at dHP and vHP, respectively). RSP, retrosplenial cortex; LS, lateral septum.

BOLD responses upon optogenetic activation of CA1 pyramidal neurons at the dHP or vHP do not necessarily reflect a direct anatomical connection. BOLD responses in the RSP upon optogenetic stimulation at the dHP, but not vHP, coincide well with anatomical evidence that the RSP receives much denser projections from CA1 pyramidal neurons at the dHP than at the vHP [9]. However, only vHP activation resulted in BOLD responses in the LS although the LS receives similar amounts of afferents from both the dHP and vHP [8]. Moreover, BOLD responses were also observed at the DG and CA3, with no direct axonal projections from CA1 pyramidal neurons. Hence, fMRI responses should have been induced through multiple direct and/or indirect synaptic connections, which might be supported by our observation that negative peak of LFP response at the DG upon the optogenetic stimulation delayed ∼18 ms from that at pyramidal cell layer of CA1 (S2A Fig.).

The exact reason for lack of BOLD responses at some brain regions with anatomical connection from CA1 pyramidal neurons, for example from dHP and vHP to LS and basomedial amygdala, respectively, remains to be elucidated. Optogenetic activation of CA1 pyramidal neurons might lead to activation of long-range GABAergic projection neurons in the hippocampus or GABAergic interneurons at the target regions [22]. More intriguing scenario is the presence of dynamical processes such that only specific patterns of activity, which were not reproduced by the current optogenetic manipulation, could propagate to some target regions [23,24]. Frequency-dependent spatial patterns of BOLD signal response upon electrical stimulation of the performant pathway, the major input to the hippocampus connecting the entorhinal cortex to the dentate gyrus, have been reported [25–27]. The electrical stimulation with 10 Hz but not with 4 or 20 Hz induced BOLD response at LS [26]. In addition, parsimonious explanation for the lack of BOLD signals would be insufficient detection sensitivity because synaptic and spiking responses of neurons in LS and amygdala have been demonstrated upon electrophysiological stimulation of dorsal and ventral hippocampal formation, respectively [28,29].

Connection strengths from the dHP or vHP to other brain areas were estimated with the amplitude of the BOLD signal change. The relationship of neuronal activity and BOLD response amplitude is not thoroughly understood [30]. In our study, peak amplitude of BOLD signal response at vHP in response to vHP stimulation, i.e., B(vHP; vHP), was larger than that of B(dHP; dHP). This may reflect the more excitable nature of CA1 pyramidal neurons at the vHP as compared to the dHP [31]. We also found B(vHP; dHP) responses but not the reverse B(dHP; vHP). This may be related to previous findings suggesting that theta waves travel from the dHP to vHP along the longitudinal axis of the hippocampus [32].

Direct optical activation of RSP or LS during light illumination at the dHP or vHP, respectively, is unlikely considering the facts that 1) expression level of ChR2(C128S) at RSP and LS is significantly lower than that at the hippocampus (Fig. 1A) [11], 2) strong expression level of ChR2(C128S) was necessary for *in vivo* optogenetic control of neurons [11], 3) significant BOLD signal response was not observed even in the dHP when light power was reduced to 10% or 20% of the original light power (S5A,B Fig.), 4) light power decreases in the brain with increasing distance from a tip of an optical fiber [33], 5) estimated light power at LS is ∼0.04% of that at the vHP (see materials and methods), 6) Light power at RSP should have been significantly lower than that at dHP although distance from optical fiber to RSP is comparable to the dHP, because RSP was located above a side of an optical fiber (Fig. 1B), whose side was painted in black to suppress stray light (Fig. 3A).

We generated transgenic mice whose CA1 pyramidal neurons express ChR2(C128S). This offers three important advantages. The first is the cell-specific expression of ChR2. A previous ofMRI study used a transgenic rat expressing ChR2 under the Thy-1.2 promoter [34]. In the study, BOLD responses may have reflected optogenetic activation beyond the hippocampus because ChR2 is expressed also in the cortex, thalamus, and hypothalamus. The second advantage is the smaller inter-subject variability in ChR2 expression as compared to the use of virus injection to mediate ChR2 expression. The third advantage is the use of a step-function opsin, ChR2(C128S), which avoids the need for continual light illumination. Pseudo-fMRI signal increases have been previously reported at the site of blue-light illumination caused by temperature-driven relaxation changes in T1 and T2* [35]. Further, comparing reported BOLD signal changes of ∼0.5% in naïve rats upon continual 30-s blue-light illumination (2 mW) to 5∼10% BOLD signal changes in our study, we conclude that heating artifacts are not primarily responsible for the effects seen. Notice potential dissociation between neuronal activation and light manipulation of the step-function opsin. Spontaneous shutdown of neuronal activities measured by LFP power or MUA was obvious (Figs. 2,3) while channels of ChR2(C128S) were open during periods between a pair of blue- and yellow-light illumination [11,36]. The step function opsin ChR2(C128S) was reported only to elevate a membrane potential to a subthreshold level instead of leading an action potential in neurons [36,37], although this effect should be dependent on the expression level of the opsin. Also notice the decrease in BOLD signal, B(RSP; vHP), upon the first yellow light illumination (the upper row of Fig. 5B and S4B Fig.), which might correspond to decreases in neuronal activities [38,39].

Recently, Weitz *et al*. reported a study employing a similar methodology as ours: measuring BOLD response upon optogenetic stimulation at the hippocampus [40]. They compared BOLD response in the brain of an anesthetized rat upon optogenetic stimulation of the dorsal and *intermediate* hippocampus which expressed virally delivered ChR2(H134R). Upon optogenetic stimulation with various frequencies such as 10, 20, 40 and 60 Hz, they observed broader BOLD responses than ours, e.g. responses at contralateral hemisphere. Their EEG recording demonstrated synchronous, seizure-like activities upon the stimulation. In our experiments, electrophysiological recording (Figs. 2,3) and *c-fos* expression staining (S6 Fig.) suggested absence of seizure activity [20]. Thus, difference in BOLD response pattern might be partly explained by the presence or absence of seizure activities in their or our study, respectively.

One limitation in our study is the use of anesthetics, which could affect neural activity, metabolism, and neurovascular coupling. Isoflurane anesthesia reduced amplitude of BOLD responses at somatosensory cortex (SI) upon electrical stimulation of hindpaw [41]. Connectivity among brain regions, which was assessed with correlation of BOLD signals, upon lentivirus-mediated optogenetic stimulation of SI was reduced by isoflurane [42]. In addition, distinct spatiotemporal pattern of resting state-fMRI was observed depending on choice of anesthetics such as isoflurane, urethane, or medetomidine [43,44]. Thus, future research using awake animals is indispensable to confirm our results [42].

Another caveat in the current study is the usage of different anesthetics, medetomidine and urethane, for ofMRI and electrophysiology, respectively. Medetomidine produces sedation by binding to α_2_-adrenoreceptors primarily located in the locus coeruleus [45], and is used frequently in fMRI studies in rodents [13,43,46]. Sedation length of a mouse by medetomidine varied from 20 to 100 min depending on its dose, and higher dose did not necessarily induce longer sedation length [13]. In our electrophysiological recording, unforeseen recovery of a mouse from anesthesia had to be avoided to prevent damage to a thin silicon probe electrode (15-μm thick). Thus we employed urethane instead of medetomidine in the electrophysiological measurements because urethane provides stable long-lasting anesthesia [47], although pharmacological mechanism of urethane is distinct from medetomidine [48]. In our fMRI experiments, sedation level was monitored with a respiration rate [13]. If a mouse recovered from sedation during the experiments, measurement was aborted and data were discarded.

Interestingly, BOLD responses at the RSP and LS upon optogenetic activation at the dHP and vHP, respectively, appear to be in good accordance with attributed functions in these regions, i.e., spatial processing at the “RSP and dHP,” and emotional responses at the “LS and vHP” [1,49,50]. These results are consistent with a contemporary memory model that suggests two separate large-scale cortical networks including “dHP and RSP” and “vHP and perirhinal cortex”, respectively [51]; the model is based on neuroanatomy, susceptibility to disease, and function. Our ofMRI data might provide additional insight into connection strength in the model.

## Supporting Information

S1 Fig Positioning of an implanted optical fiber in a transgenic mouse. Anatomical T2-weighted images showing an optical fiber implantation targetting the dHP (**A**) or vHP (**B**) of all transgenic mice used in the analysis for Figs. 4 and 5. In **B**, the brain section is rotated 11∼14 degrees laterally from the sagittal plane to depict the full length of the fiber. Animal ID is shown at upper left in each panel. Fig. 1B and C correspond to SNT061 and SNT074, respectively. Scale bar: 3 mm.
(TIF)

S2 Fig Expanded view of electrophysiological responses upon the first blue-light illumination. **A,** Local field potential (LFP) responses upon the first optogenetic stimulation averaged across animals (n = 4) is shown. Gray shading in each trace is the SEM. The blue region indicates the period of blue-light illumination used to stimulate ChR2(C128S). Red horizontal lines show average of each LFP traces prior to the stimulation. The top trace corresponds to LFP at pyramidal cell layer (pcl). Numerical values at the left (100, 500, 600, and 700) show a distance from pcl in μm. The lower 3 traces are from DG. Note that the upper 2 traces are expanded 4 times in y-axis to improve visibility. The negative-peak value (μV) and its delay time (ms) upon the blue light illumination are: −67 ± 62 μV, 26.4 ms; −61 ± 58 μV, 28.7 ms; −227 ± 234 μV, 43.2 ms; −229 ± 250, 44.0 ms; −230 ± 220 μV, 44.4 ms, respectively (from top to bottom traces). Scale bar: 0.1 mV for the upper 2 traces, 0.4 mV for the lower 3 traces.**B,** LFP spectrogram at the pcl of the CA1 region of dHP averaged across animal (n = 5) is shown. Blue and yellow triangles indicate the delivery of blue and yellow light pulses at dHP with 0.5-s duration used to activate and deactivate ChR2(C128S), respectively.
(TIF)

S3 Fig Three-dimensional statistical map of dorsal–ventral contrasts. Three-dimensional statistical map showing contrasts between activation *t*-maps of BOLD signals in response to optogenetic activation at the dHP (5 animals) and vHP (7 animals). Red and green region indicates ‘dorsal minus ventral’ and ‘ventral minus dorsal’ contrasts, respectively. These region demonstrate discordant activation upon dHP (red) and vHP (green), while Fig. 3 shows areas of overlap (yellow) upon dHP- and vHP-stimulation. The contrasts were obtained by 2nd-level random-effects analysis using two sample *t*-test (SPM8 software). Three dimensional image of the contrasts was created using Amira software (Visage Imaging, Inc.).
(TIF)

S4 Fig Expanded view of BOLD signal response upon the first blue-light illumination. BOLD signal response upon the first optogenetic activation of CA1 pyramidal neurons at the dHP (A) or vHP (B) is shown. These traces in A and B are the same as that of Fig. 5A and B, respectively. Pairs of blue and yellow vertical lines indicate periods of optogenetic activation (0.5 s duration). The x-axis at the top shows to the scan number of fMRI measurements. Grey shading indicates the SEM. The positive peak value (mean ± SEM, %) and its delay time (s) upon the blue light illumination are: 6.1 ± 1.0%, 26.3 s at dHP, 6.3 ± 1.4%, 24.8 s at retrosplenial cortex (RSP), 3.6 ± 1.7%, 6.8 s at vHP, 1.1 ± 0.4%, 5.3 s at lateral septum (LS) upon optogenetic stimulation at dHP (A); 1.0 ± 0.3%, 2.3 s at dHP, 1.8 ± 0.6%, 2.3 s at RSP, 10.9 ± 0.7%, 23.3 s at vHP, 2.1 ± 0.3%, 17.3 s at LS upon the stimulation at vHP (B).
(TIF)

S5 Fig BOLD signal at the dorsal hippocampus upon optogenetic stimulation with reduced light power or only yellow light. **A,** Time course of BOLD signal responses at the dHP upon optogenetic activation of CA1 pyramidal neurons at the dHP with 0.5 s blue and yellow light illumination separated by 30 s. Only in this measurements, light power was modulated to 10%, 20%, and 100% of that used in other experiments (i.e. Figs. 2∼4). The data are obtained from 5 measurements from 3 transgenic animals. Grey shading indicates the SEM. **B,** BOLD signal response upon optogenetic stimulation with different light power were compared using peak values of BOLD signal amplitudes during optogenetically activated periods between a pair of blue and yellow vertical lines (30 s). “pre” is the 9 s period just before the first light activation. Only 100% light power, which is used in all the experiments except this measurements, induced significant increase of BOLD signals in the dHP. **C,** Time course of BOLD signal fluctuation at the dHP upon illumination of pairs of 0.5 s yellow lights separated by 30 s at the dHP. Grey shading indicates the SEM. **D,** Time course of BOLD signal response at the dHP upon blue- and yellow-light illumination at dHP of WT mice, demonstrating absence of BOLD responses. Grey shading indicates the SEM.
(TIF)

S6 Fig Lack of *c-fos* mRNA expression upon optogenetic stimulation at dorsal or ventral hippocampus. *In situ* hybridization for *c-fos* mRNA was performed after fMRI measurement with optogenetic stimulation at the dorsal (A) or ventral (B) hippocampus (n = 5 and 7 animals, respectively). Upper and lower rows represent slices around AP −2.0 and −3.0 mm, respectively. Note lack of *c-fos* expression (no blue-purple signal) at the hippocampus, suggesting that seizure activity was not induced upon optogenetic stimulation in our condition [20]. Animals were perfused with 4% PFA ∼30 min after fMRI measurement. Scale bar: 1 mm.
(TIF)

## References

[1] Fanselow MS, Dong H-W. Are the dorsal and ventral hippocampus functionally distinct structures? Neuron. 2010;65: 7–19. 10.1016/j.neuron.2009.11.031 20152109 PMC2822727

[2] Moser E, Moser MB, Andersen P. Spatial learning impairment parallels the magnitude of dorsal hippocampal lesions, but is hardly present following ventral lesions. J Neurosci. 1993;13: 3916–3925. 8366351 10.1523/JNEUROSCI.13-09-03916.1993 PMC6576447

[3] Hughes KR. Dorsal and ventral hippocampus lesions and maze learning: influence of preoperative environment. Can J Psychol. 1965;19: 325–332. 5847750 10.1037/h0082915

[4] Kjelstrup KG, Tuvnes FA, Steffenach H-A, Murison R, Moser EI, Moser M-B. Reduced fear expression after lesions of the ventral hippocampus. Proc Natl Acad Sci USA. 2002;99: 10825–10830. 10.1073/pnas.152112399 12149439 PMC125057

[5] Ferbinteanu J, Ray C, McDonald RJ. Both dorsal and ventral hippocampus contribute to spatial learning in Long–Evans rats. Neuroscience Letters. 2003;345: 131–135. 10.1016/S0304-3940(03)00473-7 12821188

[6] Rudy JW, Matus-Amat P. The ventral hippocampus supports a memory representation of context and contextual fear conditioning: implications for a unitary function of the hippocampus. Behav Neurosci. 2005;119: 154–163. 10.1037/0735-7044.119.1.154 15727521

[7] Jung MW, Wiener SI, McNaughton BL. Comparison of spatial firing characteristics of units in dorsal and ventral hippocampus of the rat. J Neurosci. 1994;14: 7347–7356. 7996180 10.1523/JNEUROSCI.14-12-07347.1994 PMC6576902

[8] Cenquizca LA, Swanson LW. Analysis of direct hippocampal cortical field CA1 axonal projections to diencephalon in the rat. The Journal of Comparative Neurology. 2006;497: 101–114. 10.1002/cne.20985 16680763 PMC2570652

[9] Cenquizca LA, Swanson LW. Spatial organization of direct hippocampal field CA1 axonal projections to the rest of the cerebral cortex. Brain Research Reviews. 2007;56: 1–26. 10.1016/j.brainresrev.2007.05.002 17559940 PMC2171036

[10] Lee JH, Durand R, Gradinaru V, Zhang F, Goshen I, Kim D-S, et al Global and local fMRI signals driven by neurons defined optogenetically by type and wiring. Nature. 2010;465: 788–792. 10.1038/nature09108 20473285 PMC3177305

[11] Tanaka KF, Matsui K, Sasaki T, Sano H, Sugio S, Fan K, et al Expanding the repertoire of optogenetically targeted cells with an enhanced gene expression system. Cell Rep. 2012;2: 397–406. 10.1016/j.celrep.2012.06.011 22854021

[12] Baltes C, Bosshard S, Mueggler T, Ratering D, Rudin M. Increased blood oxygen level-dependent (BOLD) sensitivity in the mouse somatosensory cortex during electrical forepaw stimulation using a cryogenic radiofrequency probe. NMR Biomed. 2011;24: 439–446. 10.1002/nbm.1613 22945293

[13] Adamczak JM, Farr TD, Seehafer JU, Kalthoff D, Hoehn M. High field BOLD response to forepaw stimulation in the mouse. Neuroimage. 2010;51: 704–712. 10.1016/j.neuroimage.2010.02.083 20211267

[14] Seki F, Hikishima K, Nambu S, Okanoya K, Okano HJ, Sasaki E, et al Multidimensional MRI-CT atlas of the naked mole-rat brain (Heterocephalus glaber). Front Neuroanat. 2013;7: 45 10.3389/fnana.2013.00045 24391551 PMC3868886

[15] Song X-W, Dong Z-Y, Long X-Y, Li S-F, Zuo X-N, Zhu C-Z, et al REST: a toolkit for resting-state functional magnetic resonance imaging data processing. PLoS ONE. 2011;6: e25031 10.1371/journal.pone.0025031 21949842 PMC3176805

[16] Franklin KBJ, Paxinos G. The mouse brain in stereotaxic coordinates Amsterdam: Boston: Elsevier/Academic Press; 2008.

[17] Buzsáki G, Buhl DL, Harris KD, Csicsvari J, Czéh B, Morozov A. Hippocampal network patterns of activity in the mouse. Neuroscience. 2003;116: 201–211. 10.1016/S0306-4522(02)00669-3 12535953

[18] Quiroga RQ, Nadasdy Z, Ben-Shaul Y. Unsupervised spike detection and sorting with wavelets and superparamagnetic clustering. Neural Comput. 2004;16: 1661–1687. 10.1162/089976604774201631 15228749

[19] Stark E, Roux L, Eichler R, Senzai Y, Royer S, Buzsáki G. Pyramidal Cell-Interneuron Interactions Underlie Hippocampal Ripple Oscillations. Neuron. 2014;83: 467–480. 10.1016/j.neuron.2014.06.023 25033186 PMC4393648

[20] Morgan JI, Cohen DR, Hempstead JL, Curran T. Mapping patterns of c-fos expression in the central nervous system after seizure. Science. 1987;237: 192–197. 10.1126/science.3037702 3037702

[21] Cowansage KK, Shuman T, Dillingham BC, Chang A, Golshani P, Mayford M. Direct Reactivation of a Coherent Neocortical Memory of Context. Neuron. 2014; 10.1016/j.neuron.2014.09.022 PMC4372249 25308330

[22] Jinno S. Structural organization of long-range GABAergic projection system of the hippocampus. Front Neuroanat. 2009;3: 13 10.3389/neuro.05.013.2009 19649167 PMC2718779

[23] Buzsáki G, Geisler C, Henze DA, Wang X-J. Interneuron Diversity series: Circuit complexity and axon wiring economy of cortical interneurons. Trends Neurosci. 2004;27: 186–193. 10.1016/j.tins.2004.02.007 15046877

[24] Chaudhury D, Walsh JJ, Friedman AK, Juarez B, Ku SM, Koo JW, et al Rapid regulation of depression-related behaviours by control of midbrain dopamine neurons. Nature. 2013;493: 532–536. 10.1038/nature11713 23235832 PMC3554860

[25] Angenstein F, Kammerer E, Niessen HG, Frey JU, Scheich H, Frey S. Frequency-dependent activation pattern in the rat hippocampus, a simultaneous electrophysiological and fMRI study. NeuroImage. 2007;38: 150–163. 10.1016/j.neuroimage.2007.07.022 17728153

[26] Canals S, Beyerlein M, Murayama Y, Logothetis NK. Electric stimulation fMRI of the perforant pathway to the rat hippocampus. Magn Reson Imaging. 2008;26: 978–986. 10.1016/j.mri.2008.02.018 18479870

[27] Canals S, Beyerlein M, Merkle H, Logothetis NK. Functional MRI Evidence for LTP-Induced Neural Network Reorganization. Current Biology. 2009;19: 398–403. 10.1016/j.cub.2009.01.037 19230667

[28] McLennan H, Miller JJ. The hippocampal control of neuronal discharges in the septum of the rat. J Physiol (Lond). 1974;237: 607–624. 4596602 10.1113/jphysiol.1974.sp010500 PMC1350907

[29] Mello LEAM, Tan AM, Finch DM. Convergence of projections from the rat hippocampal formation, medial geniculate and basal forebrain onto single amygdaloid neurons: an in vivo extra- and intracellular electrophysiological study. Brain Research. 1992;587: 24–40. 10.1016/0006-8993(92)91425-E 1525648

[30] Huttunen JK, Gröhn O, Penttonen M. Coupling between simultaneously recorded BOLD response and neuronal activity in the rat somatosensory cortex. NeuroImage. 2008;39: 775–785. 10.1016/j.neuroimage.2007.06.042 17964186

[31] Dougherty KA, Islam T, Johnston D. Intrinsic excitability of CA1 pyramidal neurones from the rat dorsal and ventral hippocampus. J Physiol (Lond). 2012;590: 5707–5722. 10.1113/jphysiol.2012.242693 22988138 PMC3528986

[32] Patel J, Fujisawa S, Berényi A, Royer S, Buzsáki G. Traveling Theta Waves along the Entire Septotemporal Axis of the Hippocampus. Neuron. 2012;75: 410–417. 10.1016/j.neuron.2012.07.015 22884325 PMC3427387

[33] Stark E, Koos T, Buzsáki G. Diode probes for spatiotemporal optical control of multiple neurons in freely moving animals. Journal of Neurophysiology. 2012;108: 349–363. 10.1152/jn.00153.2012 22496529 PMC3434617

[34] Abe Y, Sekino M, Terazono Y, Ohsaki H, Fukazawa Y, Sakai S, et al Opto-fMRI analysis for exploring the neuronal connectivity of the hippocampal formation in rats. Neurosci Res. 2012;74: 248–255. 10.1016/j.neures.2012.08.007 22982343

[35] Christie IN, Wells JA, Southern P, Marina N, Kasparov S, Gourine AV, et al fMRI response to blue light delivery in the naïve brain: Implications for combined optogenetic fMRI studies. Neuroimage. 2012;66: 634–641. 10.1016/j.neuroimage.2012.10.074 23128081

[36] Berndt A, Yizhar O, Gunaydin LA, Hegemann P, Deisseroth K. Bi-stable neural state switches. Nat Neurosci. 2009;12: 229–234. 10.1038/nn.2247 19079251

[37] Boyden ES, Zhang F, Bamberg E, Nagel G, Deisseroth K. Millisecond-timescale, genetically targeted optical control of neural activity. Nat Neurosci. 2005;8: 1263–1268. 10.1038/nn1525 16116447

[38] Van Zijl PCM, Hua J, Lu H. The BOLD post-stimulus undershoot, one of the most debated issues in fMRI. NeuroImage. 2012;62: 1092–1102. 10.1016/j.neuroimage.2012.01.029 22248572 PMC3356682

[39] Shmuel A, Augath M, Oeltermann A, Logothetis NK. Negative functional MRI response correlates with decreases in neuronal activity in monkey visual area V1. Nat Neurosci. 2006;9: 569–577. 10.1038/nn1675 16547508

[40] Weitz AJ, Fang Z, Lee HJ, Fisher RS, Smith WC, Choy M, et al Optogenetic fMRI reveals distinct, frequency-dependent networks recruited by dorsal and intermediate hippocampus stimulations. NeuroImage. 2015;107: 229–241. 10.1016/j.neuroimage.2014.10.039 25462689 PMC4409430

[41] Nair G, Duong TQ. Echo-planar BOLD fMRI of mice on a narrow-bore 9.4 T magnet. Magn Reson Med. 2004;52: 430–434. 10.1002/mrm.20158 15282829 PMC2949950

[42] Desai M, Kahn I, Knoblich U, Bernstein J, Atallah H, Yang A, et al Mapping Brain Networks in Awake Mice Using Combined Optical Neural Control and fMRI. J Neurophysiol. 2011;105: 1393–1405. 10.1152/jn.00828.2010 21160013 PMC3074423

[43] Grandjean J, Schroeter A, Batata I, Rudin M. Optimization of anesthesia protocol for resting-state fMRI in mice based on differential effects of anesthetics on functional connectivity patterns. Neuroimage. 2014;102P2: 838–847. 10.1016/j.neuroimage.2014.08.043 25175535

[44] Jonckers E, Delgado Y Palacios R, Shah D, Guglielmetti C, Verhoye M, Van der Linden A. Different anesthesia regimes modulate the functional connectivity outcome in mice. Magn Reson Med. 2013;72: 1103–1112. 10.1002/mrm.24990 24285608

[45] Sinclair MD. A review of the physiological effects of alpha2-agonists related to the clinical use of medetomidine in small animal practice. Can Vet J. 2003;44: 885–897. 14664351 PMC385445

[46] Weber R, Ramos-Cabrer P, Wiedermann D, van Camp N, Hoehn M. A fully noninvasive and robust experimental protocol for longitudinal fMRI studies in the rat. Neuroimage. 2006;29: 1303–1310. 10.1016/j.neuroimage.2005.08.028 16223588

[47] Takata N, Shinohara Y, Ohkura M, Mishima T, Nakai J, Hirase H. Imaging of Astrocytic Activity in Living Rodents In: Weber B, Helmchen F, editors. Optical Imaging of Neocortical Dynamics. Springer Berlin / Heidelberg, in press; 2014 pp. 191–207.

[48] Hara K, Harris RA. The anesthetic mechanism of urethane: the effects on neurotransmitter-gated ion channels. Anesth Analg. 2002;94: 313–318, table of contents. 11812690 10.1097/00000539-200202000-00015

[49] Vann SD, Aggleton JP, Maguire EA. What does the retrosplenial cortex do? Nat Rev Neurosci. 2009;10: 792–802. 10.1038/nrn2733 19812579

[50] Singewald GM, Rjabokon A, Singewald N, Ebner K. The Modulatory Role of the Lateral Septum on Neuroendocrine and Behavioral Stress Responses. Neuropsychopharmacology. 2011;36: 793–804. 10.1038/npp.2010.213 21160468 PMC3055728

[51] Ranganath C, Ritchey M. Two cortical systems for memory-guided behaviour. Nat Rev Neurosci. 2012;13: 713–726. 10.1038/nrn3338 22992647

