# Supplementary figures and images for: Optogenetic Activation of CA1 Pyramidal Neurons at the Dorsal and Ventral Hippocampus Evokes Distinct Brain-Wide Responses Revealed by Mouse fMRI
Source: PLoS One. 2015 Mar 20;10(3):e0121417. doi: 10.1371/journal.pone.0121417 (PMC4368201; doi:10.1371/journal.pone.0121417)

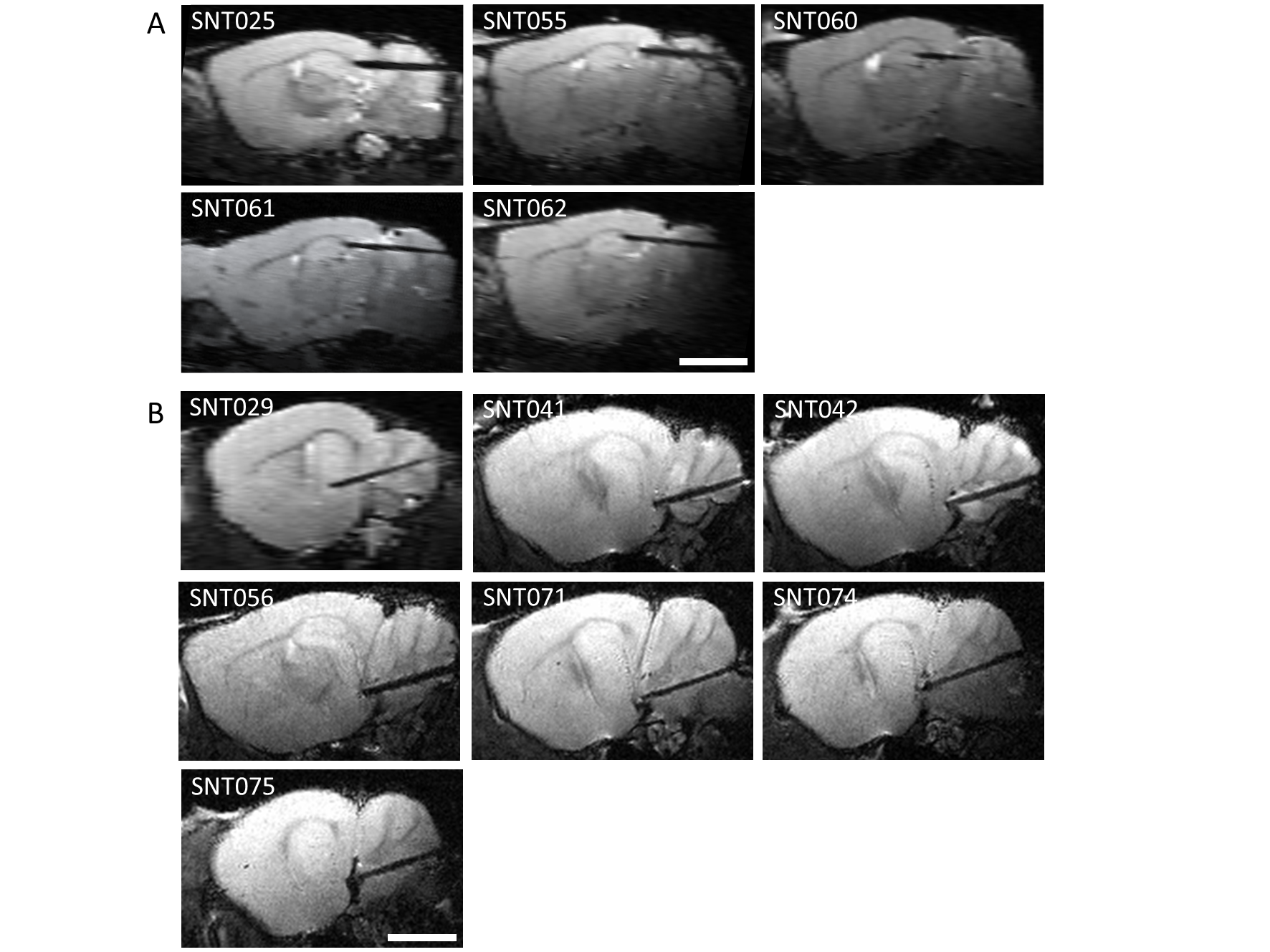

Supplement: S1 Fig — Anatomical T2-weighted images showing an optical fiber implantation targetting the dHP (A) or vHP (B) of all transgenic mice used in the analysis for Figs. 4 and 5. In B, the brain section is rotated 11∼14 degrees laterally from the sagittal plane to depict the full length of the fiber. Animal ID is shown at upper left in each panel. Fig. 1B and C correspond to SNT061 and SNT074, respectively. Scale bar: 3 mm. (TIF) [file pone.0121417.s001.tif]

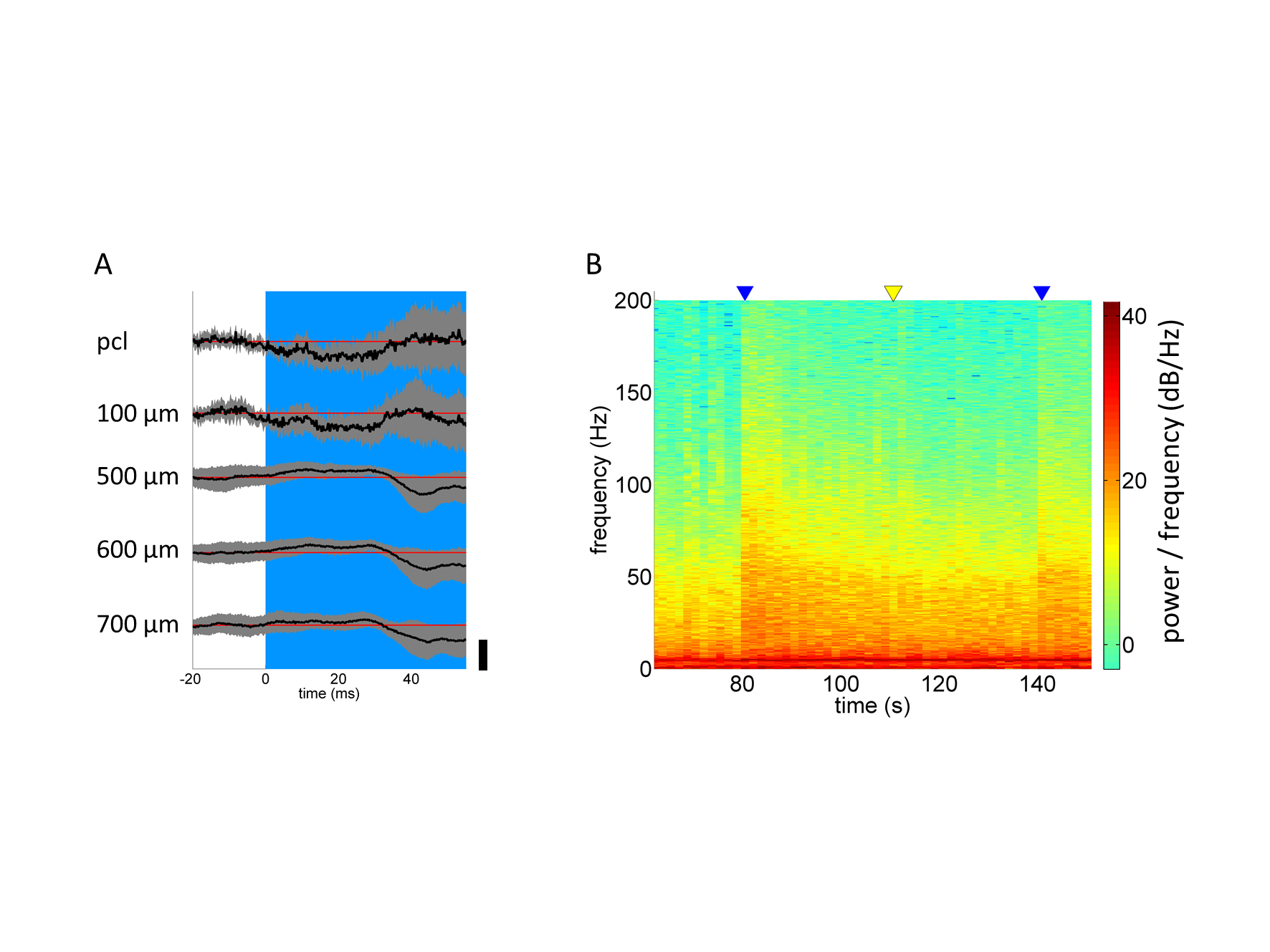

Supplement: S2 Fig — A, Local field potential (LFP) responses upon the first optogenetic stimulation averaged across animals (n = 4) is shown. Gray shading in each trace is the SEM. The blue region indicates the period of blue-light illumination used to stimulate ChR2(C128S). Red horizontal lines show average of each LFP traces prior to the stimulation. The top trace corresponds to LFP at pyramidal cell layer (pcl). Numerical values at the left (100, 500, 600, and 700) show a distance from pcl in μm. The lower 3 traces are from DG. Note that the upper 2 traces are expanded 4 times in y-axis to improve visibility. The negative-peak value (μV) and its delay time (ms) upon the blue light illumination are: −67 ± 62 μV, 26.4 ms; −61 ± 58 μV, 28.7 ms; −227 ± 234 μV, 43.2 ms; −229 ± 250, 44.0 ms; −230 ± 220 μV, 44.4 ms, respectively (from top to bottom traces). Scale bar: 0.1 mV for the upper 2 traces, 0.4 mV for the lower 3 traces.B, LFP spectrogram at the pcl of the CA1 region of dHP averaged across animal (n = 5) is shown. Blue and yellow triangles indicate the delivery of blue and yellow light pulses at dHP with 0.5-s duration used to activate and deactivate ChR2(C128S), respectively. (TIF) [file pone.0121417.s002.tif]

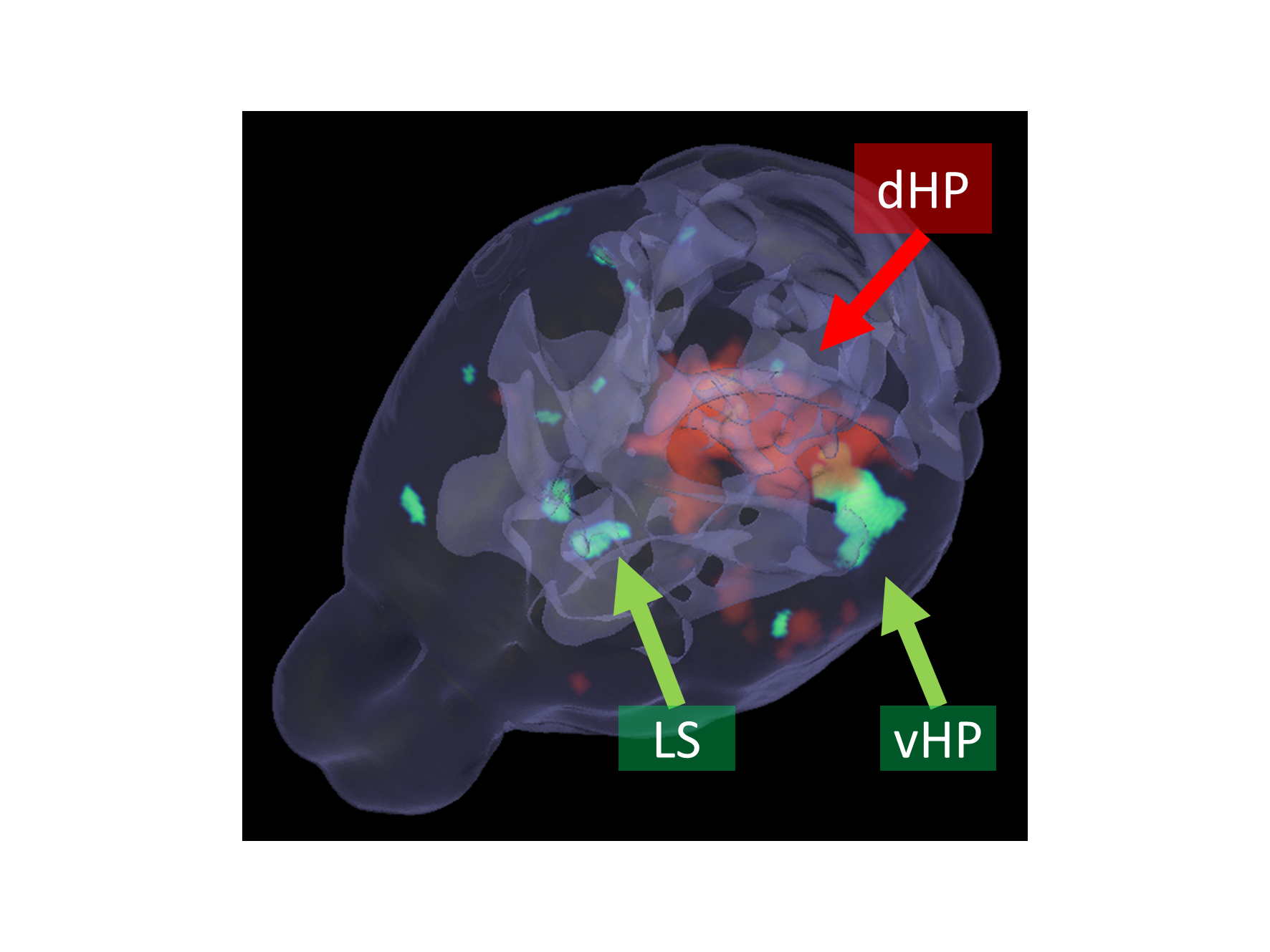

Supplement: S3 Fig — Three-dimensional statistical map showing contrasts between activation t-maps of BOLD signals in response to optogenetic activation at the dHP (5 animals) and vHP (7 animals). Red and green region indicates ‘dorsal minus ventral’ and ‘ventral minus dorsal’ contrasts, respectively. These region demonstrate discordant activation upon dHP (red) and vHP (green), while Fig. 3 shows areas of overlap (yellow) upon dHP- and vHP-stimulation. The contrasts were obtained by 2nd-level random-effects analysis using two sample t-test (SPM8 software). Three dimensional image of the contrasts was created using Amira software (Visage Imaging, Inc.). (TIF) [file pone.0121417.s003.tif]

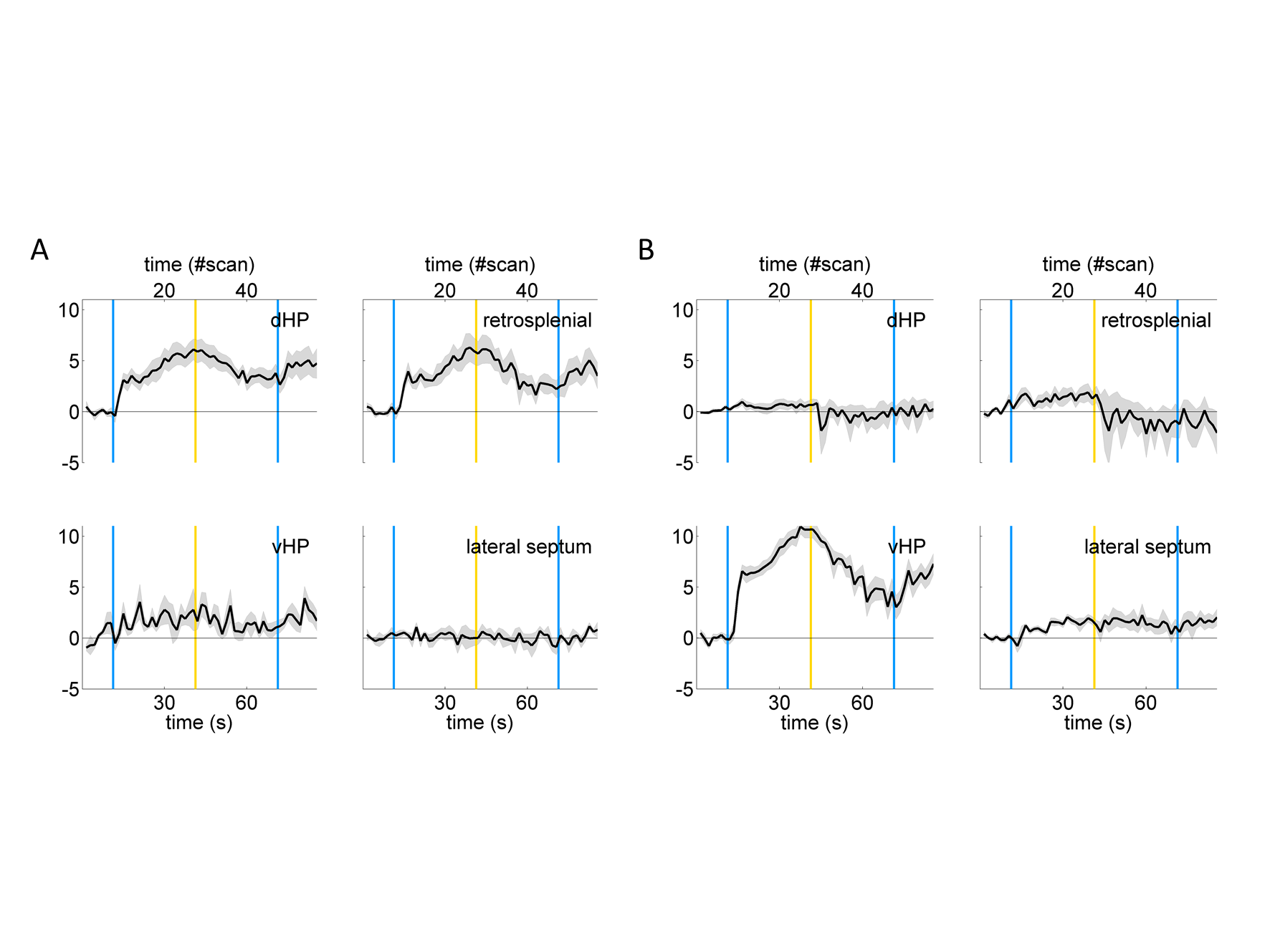

Supplement: S4 Fig — BOLD signal response upon the first optogenetic activation of CA1 pyramidal neurons at the dHP (A) or vHP (B) is shown. These traces in A and B are the same as that of Fig. 5A and B, respectively. Pairs of blue and yellow vertical lines indicate periods of optogenetic activation (0.5 s duration). The x-axis at the top shows to the scan number of fMRI measurements. Grey shading indicates the SEM. The positive peak value (mean ± SEM, %) and its delay time (s) upon the blue light illumination are: 6.1 ± 1.0%, 26.3 s at dHP, 6.3 ± 1.4%, 24.8 s at retrosplenial cortex (RSP), 3.6 ± 1.7%, 6.8 s at vHP, 1.1 ± 0.4%, 5.3 s at lateral septum (LS) upon optogenetic stimulation at dHP (A); 1.0 ± 0.3%, 2.3 s at dHP, 1.8 ± 0.6%, 2.3 s at RSP, 10.9 ± 0.7%, 23.3 s at vHP, 2.1 ± 0.3%, 17.3 s at LS upon the stimulation at vHP (B). (TIF) [file pone.0121417.s004.tif]

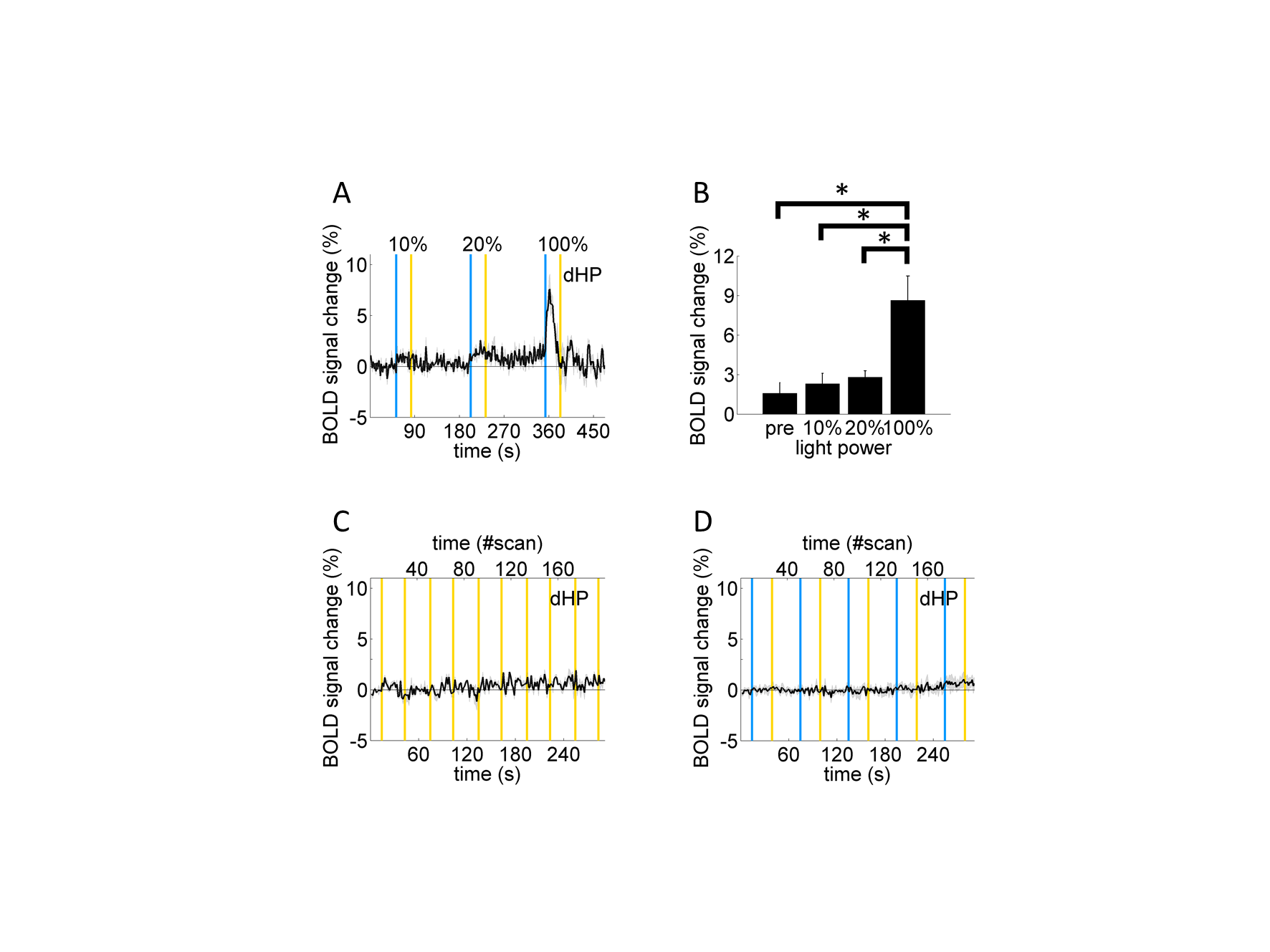

Supplement: S5 Fig — A, Time course of BOLD signal responses at the dHP upon optogenetic activation of CA1 pyramidal neurons at the dHP with 0.5 s blue and yellow light illumination separated by 30 s. Only in this measurements, light power was modulated to 10%, 20%, and 100% of that used in other experiments (i.e. Figs. 2∼4). The data are obtained from 5 measurements from 3 transgenic animals. Grey shading indicates the SEM. B, BOLD signal response upon optogenetic stimulation with different light power were compared using peak values of BOLD signal amplitudes during optogenetically activated periods between a pair of blue and yellow vertical lines (30 s). “pre” is the 9 s period just before the first light activation. Only 100% light power, which is used in all the experiments except this measurements, induced significant increase of BOLD signals in the dHP. C, Time course of BOLD signal fluctuation at the dHP upon illumination of pairs of 0.5 s yellow lights separated by 30 s at the dHP. Grey shading indicates the SEM. D, Time course of BOLD signal response at the dHP upon blue- and yellow-light illumination at dHP of WT mice, demonstrating absence of BOLD responses. Grey shading indicates the SEM. (TIF) [file pone.0121417.s005.tif]

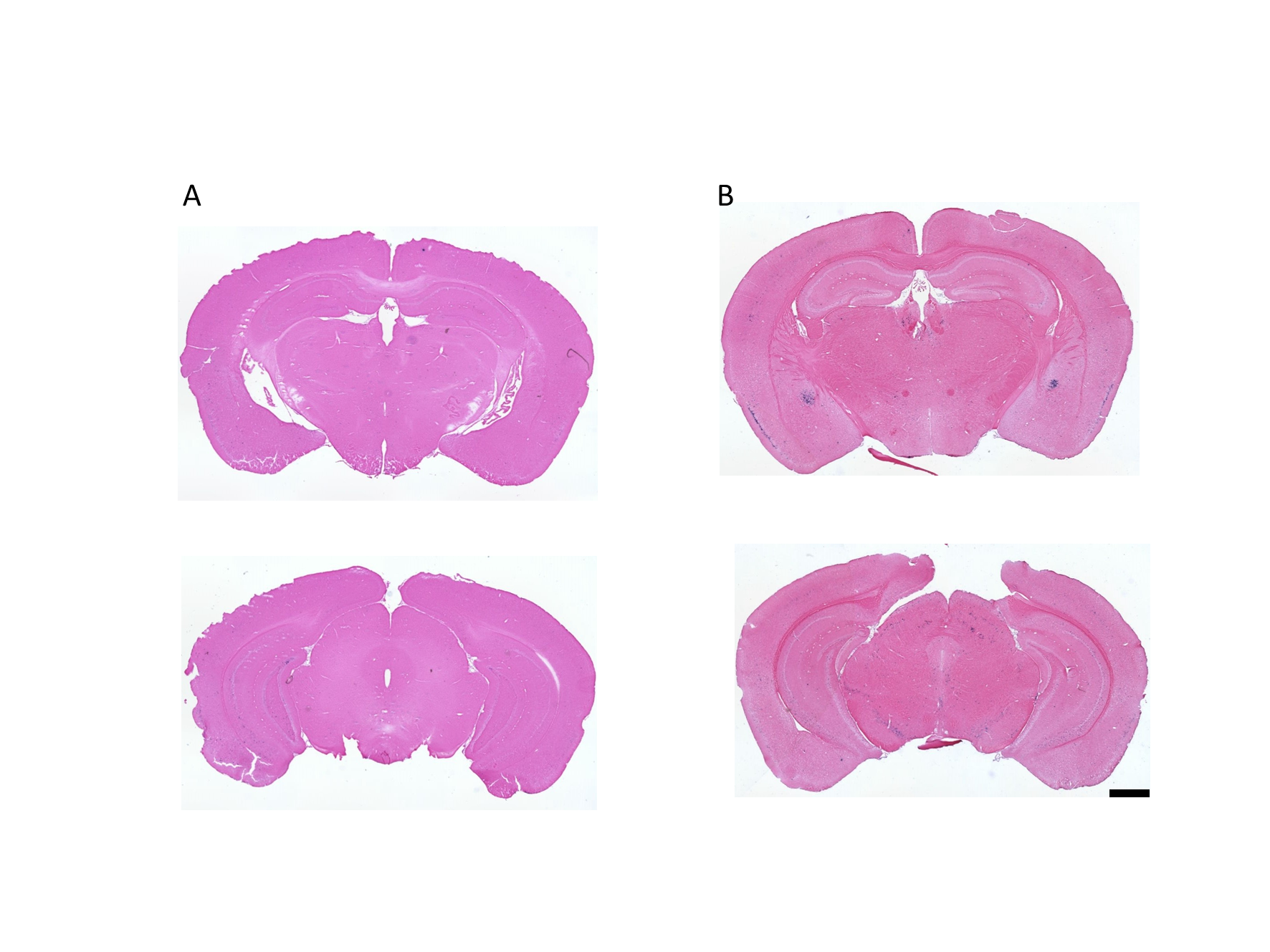

Supplement: S6 Fig — In situ hybridization for c-fos mRNA was performed after fMRI measurement with optogenetic stimulation at the dorsal (A) or ventral (B) hippocampus (n = 5 and 7 animals, respectively). Upper and lower rows represent slices around AP −2.0 and −3.0 mm, respectively. Note lack of c-fos expression (no blue-purple signal) at the hippocampus, suggesting that seizure activity was not induced upon optogenetic stimulation in our condition [20]. Animals were perfused with 4% PFA ∼30 min after fMRI measurement. Scale bar: 1 mm. (TIF) [file pone.0121417.s006.tif]
